# Gastrosplenic fistula due to splenic lymphoma: two case reports and review of the literature

**DOI:** 10.1186/s13256-024-04441-2

**Published:** 2024-03-07

**Authors:** Feryel Letaief Ksontini, Yosra Zaimi, Isaad Nefzi, Salim Khrouf, Myriam Ayari, Sonia Sghaier, Asma Zidi, Houcine Magherbi, Mouna Ayadi

**Affiliations:** 1 Department of Medical Oncology, Salah Azaiez Institute, Tunis El Manar University, Tunis, Tunisia; 2 Department of Gastroenterology, Charles Nicolle Hospital, Tunis El Manar University, Tunis, Tunisia; 3 Department of Surgery A, La Rabta Hospital, Tunis El Manar University, Tunis, Tunisia; 4 Department of Radiology, Salah Azaiez Institute, Tunis El Manar University, Tunis, Tunisia

**Keywords:** Lymphoma, Gastrosplenic fistula, Chemotherapy, Surgery

## Abstract

**Background:**

Gastrosplenic fistula is a rare and potentially fatal complication of various conditions. Lymphoma is the most common cause. It can occur spontaneously or after chemotherapy. Gastrosplenic fistula diagnosis can be confused with a splenic abscess because of the presence of air into the mass. The computed tomography identification of the fistulous tract is the key to a right diagnosis. Treatment modalities include surgical resection, chemotherapy, or a combination of both.

**Case presentation:**

Here we report two patients with gastrosplenic fistula due to diffuse large B cell lymphoma. The first patient was a 54-year-old Caucasian woman with an enormous primary splenic diffuse large B cell lymphoma leading to the development of a spontaneous fistula in the stomach. The second patient was a 48-year-old Caucasian male patient with an enormous splenic diffuse large B cell lymphoma complicated by fistula after chemotherapy. Both patients died of septic shock several days after surgery.

**Conclusion:**

Gastrosplenic fistula is a rare complication with a poor-prognosis, for which surgery is currently the preferred treatment.

## Introduction

Gastrosplenic fistula (GSF) is a rare manifestation of splenic [1, 2] or stomach lesions [3]. In most cases, this uncommon complication is due to diffuse large B cell lymphoma (DLBC) [4] of the spleen due to the aggressive nature of this tumor leading to gastric wall invasion. Hodgkin’s lymphoma represents the second most frequent GSF etiology, followed by histiocytic lymphoma. It can occur spontaneously or after chemotherapy. The management of these lesions is still challenging. Here, we report two cases of GSF complicating a splenic DLBC lymphoma. The first case is a spontaneous fistula, and the second one developed during chemotherapeutic treatment of the underlying DLBC.

## Case presentation

### Case 1

A 54-year-old-Caucasian-woman presented at the emergency room with a 1-month history of left upper quadrant abdominal pain, constipation, fatigue, loss of weight, and vomiting for 3 days.

The physical examination showed a patient in otherwise good general health. She presented left hypochondrium pain with a 13cm splenomegaly with no hepatomegaly or palpable peripheral lymphadenopathy.

Laboratory studies revealed only hypochromic microcytic anemia (hemoglobin: 8.3 g/dl). A thoraco-abdominal computed tomography (CT) revealed a splenic mass measuring 13.5 cm adherent to the stomach and intra-abdominal and retroperitoneal lymphadenopathy.

**Fig. 1:**
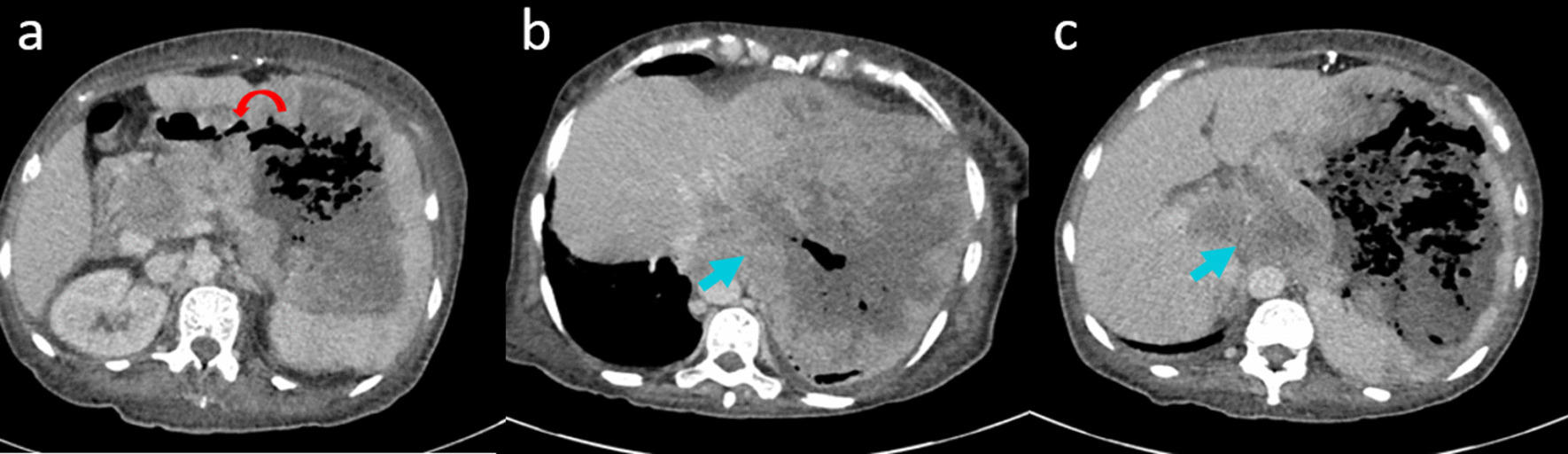
Abdominal CT scan views. **a** A gas-containing splenic mass due to the fistulization in the stomach (red arrow) **b**, **c** retroperitoneal lymphadenopathy (blue arrow)

Trans-parietal biopsy was not considered safe, because of the hemorrhagic risk. Therefore, we performed a laparoscopic surgical biopsy to reach a diagnosis. Intraoperative findings revealed a locally advanced large splenic mass invading the stomach and adhering to left lobe of the liver. This mass also invaded the left diaphragm and compressed the pancreas. As it was unresectable, biopsy was performed. Histopathology showed a to diffuse large B cell lymphoma positive for CD20, Bcl6, Bcl2, and Ki67: 90%. We completed investigations with a positron emission tomography (PET) scan that showed an active lymphatic splenic lesion with lymphadenopathy above and under the diaphragm. The tumor was classified stage III, and the patient was to start chemotherapy within a few days. However, she presented after 2 days at our hospital with an altered mental state and abdominal pain. A CT scan revealed a rapid progression with a 21 cm necrotic mass of the upper spleen pole, with air within the splenic mass corresponding to multiple gastrosplenic fistula with the gastric corpus (Fig. 1).

The largest fistula measured 1 cm and connected the mass with left wall of the stomach. A gastrectomy with splenectomy was planned. Unfortunately, the patient presented with septic shock and died.

### Case 2

A 48-year-old Caucasian male patient with no past medical history, presented at our hospital with chest pain, dyspnea, fatigue, and weight loss. Physical examination revealed bilateral cervical nodes of 3 cm, a left axillary node of 5 cm, and a splenomegaly of 15 cm. The patient had anemia (hemoglobin of 10 g/dl), leucocytosis (19,780/mm^3^), thrombocytosis (593,000/mm^3^) with raised C-reactive protein (40 mg/l). The thoraco-abdominal CT scan revealed a pulmonary mass of the left lobe measuring 8.5 × 5.6 × 5 cm, two hilar pulmonary masses, a splenic mass of 15.5 × 13 cm, and left adrenal mass of 5.7 × 4.8 cm.

**Fig. 2:**
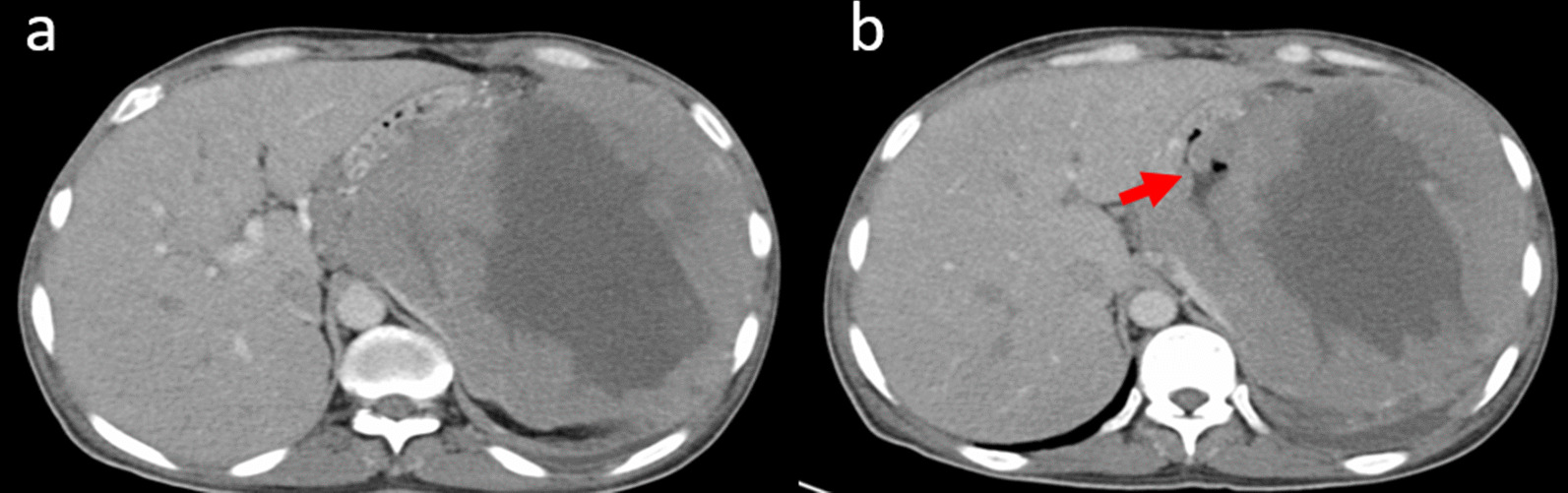
Computed tomography scan views demonstrating a large necrotic splenic mass (**a**) communicating with the stomach via a gastrosplenic fistula (**b**, red arrow)

A biopsy of the axillary lymphadenectomy and of the pulmonary mass led to the diagnosis of a DLBC lymphoma. Both of the two biopsies stained positive for CD20, bcl6, bcl2, and Cmyc and the Ki67 was 80%. The tumor was classified stage IV. Chemotherapy with rituximab, adriablastine, cyclophosphamide, vincristine, and prednisone (RCHOP) was initiated. After the second cycle, he presented at our emergency room for worsening of his dyspnea with abdominal pain. CT scan revealed a large 16.8 × 15.9 cm necrotic mass in the spleen communicating with the stomach via a gastrosplenic fistula with a presence of a posterior spleno-pleural fistula (Fig. 2). This mass infiltrated the greater curvature of the stomach, a part of the body, and the tail of the pancreas. The patient underwent surgery consisting of a partial gastrectomy with splenectomy and distal pancreatomy. He died 1 week after the surgery of septic shock due to an anastomotic leak.

## Discussion

Gastrosplenic fistula is a very rare and potentially fatal condition mainly caused by lymphomas. The most frequent etiology is diffuse large B cell lymphomas. The development of this complication is secondary to the aggressive nature of this tumor leading to invasion of the adjacent organs. GSF occurs spontaneously [5, 6] or after chemotherapy [7] as a result of rapid regression of the tumor that is infiltrating the gastric mucosa, thus resulting in a fistula. These features were seen in our cases. A systemic review of lymphoma-related gastric fistulas reviewed 27 cases of spontaneous and chemotherapy induced GSF [4]. Men were more affected than women (22 cases). A total of 17 cases have been reported to be spontaneous. Most of the patients presented with abdominal pain, splenomegaly, vomiting, and fatigue. Some others presented with hematemesis or melena and epigastric pain. These clinical characteristics were also present in our patients. But in our cases the tumor became rapidly complicated.

Since evoking the diagnosis can be difficult, as the symptoms are general and nonspecific, CT scan is the key in identifying gastrosplenic fistulas [7]. Indeed, if GSF is not detected early, it can progress and involve the diaphragm. In our review of the literature, we found only a few cases reported with extensive lymphoma masses in the left upper quadrant, including diaphragm involvement similar to our case [4].

GSF diagnosis can be confused with a splenic abscess due to the presence of air in the mass. The CT identification of the fistulous tract is the key to a right diagnosis. Filling of the splenic cavity by orally administered gastrointestinal contrast medium with CT imaging, endoscopy of the upper gastrointestinal tract, and PET scan could also be helpful in cases where there doubt [8]. When performing a PET scan, the GSF can be visualized as the most hypermetabolic region in an increased activity spleen.

Primary lesion, whether splenic or gastric, may be difficult to determine at first sight, but changes in CT scan over time may clear up the situation. Imagery typically shows a necrotic mass filled with air, seen in the CT scan as air bubbles. A fistulous tract from the spleen to the gastric lumen can also be visible.

A commonly accepted condition required for GSF to happen is the rapid necrosis of infiltrated lymphoma cells in the spleen capsule. In 1984, a clinical and pathological characteristics study of 10 cases of spleen lymphomas [9] identified the distinctive DLBCL characteristic of presenting with large, destructive mass with extensive central necrosis and often capsular penetration and local invasion of adjacent organs such as the stomach, diaphragm, or pancreas. Most of described GSF cases are related to splenic mass ranging from 3.6 to 8 cm [4]. If a large part of the gastric wall is infiltrated, then a large fistula will arise as in our two cases. In our review of the literature, we could only find a couple cases of a splenic mass measuring 15 and 18 cm [4].

The management of this lesion is challenging. Surgery is the most reported treatment of GSF [10]. The most common surgical treatment is partial gastrectomy with splenectomy. For large tumors, a near total gastrectomy and splenectomy may be needed. Ineligible patients for surgery have a poor outcome. Only a few cases of GSF were reported to be treated with chemotherapy or chemotherapy followed by radiation therapy [11].

## Conclusion

In conclusion, despite the bad outcomes of our two patients with huge splenic lymphomas, splenectomy with partial gastrectomy remains the preferred treatment for an established GSF according to the literature review. Further investigations and studies should be done to better evaluate how to treat GSF without surgery.

## Data Availability

All data are available as part of the article, and no additional source data are required.

## References

[1] Ding YL, Wang SY (2012). Gastrosplenic fistula due to splenic large B-cell lymphoma. J Res Med Sci.

[2] Kapoor A, Nanavati S, Kumar V, Azam S, Komal F, Singhal M (2018). Aerosplenomegaly: case of gastrosplenic fistula and splenic abscess in lymphom. Am J Gastroenterol.

[3] Kerem M, Sakrak O, Yilmaz TU, Gultekin FA, Dursun A, Bedirli A (2006). Spontaneous gastrosplenic fistula in primary gastric lymphoma: surgical management. Asian J Surg.

[4] Kang DH, Huh J, Lee JH, Jeong YK, Cha HJ (2017). Gastrosplenic fistula occurring in lymphoma patients: systematic review with a new case of extranodal NK/T-cell lymphoma. World J Gastroenterol.

[5] Choi JE, Chung HJ, Lee HG (2002). Spontaneous gastrosplenic fistula: a rare complication of splenic diffuse large cell lymphoma. Abdom Imaging.

[6] Khan F, Vessal S, McKimm E, D’Souza R (2010). Spontaneous gastrosplenic fistula secondary to primary splenic lymphoma. BMJ Case Rep.

[7] Moghazy KM (2008). Gastrosplenic fistula following chemotherapy for lymphoma. Gulf J Oncolog.

[8] Wang TP, Doss M, Tokar JL, Reddy S, Barta SK, Yu JQ (2017). Lymphoma causing gastrosplenic fistula revealed by FDG PET/CT. Clin Nucl Med.

[9] Harris NL, Aisenberg AC, Meyer JE, Ellman L, Elman A (1984). Diffuse large cell (histiocytic) lymphoma of the spleen. Clinical and pathologic characteristics of ten cases. Cancer.

[10] Rothermel LD, Chadwick CL, Thambi-Pillai T (2010). Gastrosplenic fistula: etiologies, diagnostic studies, and surgical management. Int Surg.

[11] Saito M, Miyashita K, Miura Y, Harada S, Ogasawara R, Izumiyama K (2019). Successful treatment of gastrosplenic fistula arising from diffuse large B-cell lymphoma with chemotherapy: two case reports. Case Rep Oncol.

